# Rol de enfermería frente al delirium en unidad de cuidado intensivo pediátrico: Scoping Review*

**DOI:** 10.15649/cuidarte.2381

**Published:** 2022-10-19

**Authors:** Lidia Esther Oostra Cortés, Ángela María Henao Castaño, Claudia Lorena Motta Robayo

**Affiliations:** 1 . Universidad Nacional de Colombia. Bogotá, Colombia. Email: loostrac@ unal.edu.co Universidad Nacional de Colombia Universidad Nacional de Colombia Bogotá Colombia loostrac@ unal.edu.co; 2 . Facultad de Enfermería. Universidad Nacional de Colombia. Bogotá, Colombia. Email: angmhenaocas@unal.edu.co Universidad Nacional de Colombia Facultad de Enfermería Universidad Nacional de Colombia Bogotá Colombia; 3 . Universidad Nacional de Colombia. Bogotá, Colombia. Email: clmottar@unal.edu.co Universidad Nacional de Colombia Universidad Nacional de Colombia Bogotá Colombia clmottar@unal.edu.co

**Keywords:** Delirium Pediátrico, Unidades de Cuidado Intensivo Pediátrico, Cuidado del Niño, Cuidados Críticos, Enfermeras Pediátricas, Pediatric Delirium, Pediatric Intensive Care Units, Child Care, Critical Care, Pediatric Nursing, Delirium Pediátrico, Unidades de Terapia Intensiva Pediátrica, Cuidado da Crianga, Cuidados Críticos, Enfermagem Pediátrica

## Abstract

**Introducción:**

El manejo integral de delirium tiene componentes de diferente índole y el rol de enfermería frente a éste puede ser difuso.

**Objetivo:**

Identificar en la literatura disponible los cuidados de enfermería no farmacológicos para niños hospitalizados en Unidad de Cuidado Intensivo Pediátrica que presenten delirium.

**Materiales y métodos:**

Se realizó una búsqueda estratégica en Web Of Science, Medline, Science Direct, Scielo, Biblioteca Virtual en Salud, LILACS y Open Grey utilizando los términos “nursing care”, “child OR children”, “delirium”, y “Pediatric Intensive Care Unit”. La extracción y análisis de los datos se dio por medio de una matriz.

**Resultados:**

Se identificaron 12 artículos que cumplían con los criterios de inclusión y se clasificaron en 4 categorías según la intervención principal desarrollada en el estudio: Abordaje investigativo, intervenciones de confort, intervenciones integrales, e intervenciones educativas.

**Discusión:**

El cuidado de enfermería frente al delirium comprende medidas preventivas o curativas que parten del paciente como centro y se extienden hasta su entorno y su familia. Las intervenciones de enfermería pueden estar interrelacionadas de manera que se sustentan y complementan entre ellas. Algunas actividades de cuidado pueden considerarse un indicador de calidad de la atención en salud.

**Conclusiones:**

Para abordar integralmente el delirium pediátrico es necesario incidir sobre los factores individuales, ambientales y estructurales que contribuyen a su aparición. El cuidado de enfermería frente al delirium constituye una forma de proteger y promover el bienestar y el desarrollo inmediato y futuro de los niños.

## Abstract (en)

**Introduction:**

Comprehensive management of delirium has components of different kinds, and the role of nursing in it may be unclear.

**Objective:**

To identify in the literature available non-pharmacological nursing care for children with delirium hospitalized in Pediatric Intensive Care Units.

**Materials and Methods:**

A strategic search was performed in Web of Science, MEDLINE, Science Direct, Scielo, Virtual Health Library, LILACS, and Open Grey using the terms “nursing care,” “child OR children,” “delirium,” and “Pediatric Intensive Care Unit.” Data extraction and analysis were performed using a matrix.

**Results:**

Twelve articles were identified that met the inclusion criteria and were classified into four categories according to the main intervention developed in the study: Research approach, comfort interventions, comprehensive interventions, and educational interventions.

**Discussion:**

Nursing care for delirium includes preventive or curative measures starting from the patient as the center and extending to the patient's environment and family. Nursing interventions may be interrelated in a way that supports and complements each other. Some care activities may be considered an indicator of the quality of care.

**Conclusions:**

A comprehensive approach to pediatric delirium requires addressing individual, environmental, and structural factors that contribute to its onset. Delirium nursing care is a way to protect and promote children's immediate and future well-being and development.

## Resumo

**Introdugáo:**

O manejo integral do delirium possui componentes de natureza diversa e o papel da enfermagem frente a ele pode ser difuso.

**Objetivo:**

identificar os cuidados de enfermagem nao farmacológicos na literatura disponível para changas internadas na UTI. Cuidado com delirio.

**Materiais e métodos:**

Foi realizada uma busca estratégica no Web Of Science, Medline, Science Direct, Scielo, Biblioteca Virtual em Saúde, LILACS e Open Gray utilizando os termos "cuidado de enfermagem", "crianga OR criangas", "delirium" e " Unidade de Terapia Intensiva Pediátrica”. A extragao e análise dos dados se deu por meio de uma matriz.

**Resultados:**

foram identificados 12 artigos que atenderam aos critérios de inclusao e foram classificados em 4 categorias de acordo com a principal intervengao desenvolvida no estudo: abordagem investigativa, intervengóes de conforto, intervengóes integrais e intervengóes educativas.

**Discussao:**

O cuidado de enfermagem contra o delirium inclui medidas preventivas ou curativas que partem do paciente como centro e se estendem ao seu ambiente e família. As intervengóes de enfermagem podem ser inter relacionadas de forma que se apoiem e se complementem. Algumas atividades assistenciais podem ser consideradas um indicador da qualidade da assisténcia a saúde.

**Conclusóes:**

Para abordar de forma abrangente o delirium pediátrico, é necessário focar nos fatores individuais, ambientais e estruturais que contribuem para o seu aparecimento. A assisténcia de enfermagem ao delirium é uma forma de proteger e promover o bem-estar e o desenvolvimento imediato e futuro da crianga.

## Introducción

El delirium es un trastorno neurocognitivo caracterizado por una alteración de la atención o la conciencia que se acompaña de una alteración cognitiva y no se puede explicar mejor por otra alteración neurocognitiva preexistente o en curso^1^. Las principales manifestaciones clínicas son alteraciones “de la percepción, del pensamiento, de la memoria, del comportamiento psicomotor, de la emoción y del ciclo sueño-vigilia”^2^. Se clasifica según la actividad psicomotora que presenta el paciente, de modo que puede ser hiperactivo, hipoactivo o mixto^1^^,^^3^.

La Asociación Americana de Psiquiatría en el Manual Diagnóstico y Estadístico de Trastornos Mentales (DSM-V) establece los siguientes criterios diagnósticos para el delirium^1^:

Una alteración de la atención y la conciencia,
Que aparece en poco tiempo, constituye un cambio respecto al estado inicial y tiende a fluctuar durante el día y
Una alteración cognitiva adicional.
Los criterios A y C no se explican mejor por otra alteración neurocognitiva preexistente, establecida o en curso.
En la anamnesis, exploración física o análisis clínicos se obtienen datos que indican que la alteración es una consecuencia fisiológica directa de otra afección médica, una intoxicación, una abstinencia por una sustancia, una exposición a una toxina o se debe a múltiples etiologías.

El Manual indica que se debe especificar si el delirium es desencadenado por una intoxicación, por la abstinencia de una sustancia, por un medicamento, por otra afección médica, o por múltiples etiologías^1^. Por otro lado, se considera que el delirium es el resultado de “desbalances en la síntesis, liberación e inactivación de los neurotransmisores que normalmente controlan la función cognitiva, el comportamiento y el estado de ánimo”, como la dopamina, el ácido gamma-aminobutírico (GABA) y la acetilcolina^3^. En el caso del delirium pediátrico (DP) en la Unidad de Cuidado Intensivo (UCI) este desequilibrio puede ser secundario a la enfermedad crítica, a su tratamiento y/o al entorno de la unidad^4^^,^^5^. Adicionalmente existen diferentes factores de riesgo que contribuyen al desbalance de neurotransmisores que conlleva a la aparición de delirium, entre ellos algunas condiciones preexistentes del sistema nervioso central (SNC), algunas terapias como la ventilación mecánica, las benzodiacepinas y los anticolinérgicos, factores inherentes a la UCI como el entorno desconocido u hostil, la falta de interacción social y las edad menor de 2 años^3^^,^^4^.

La estrategia por excelencia para tratamiento del delirium es identificar la causa subyacente y tratarla^3^^)^ mientras se aplican intervenciones de confort y medidas de bienestar^6^. También se pueden utilizar algunos medicamentos antipsicóticos. Pat el et al. proponen un algoritmo para el manejo de delirium compuesto por 4 dimensiones: El tratamiento de la enfermedad subyacente, el control de factores iatrogénicos, las modificaciones ambientales y el manejo farmacológico^4^.

A pesar de ser una entidad transitoria, el DP tiene potencial para desencadenar resultados adversos en los niños tales como deterioro físico, funcional, neurocognitivo y psicológico transitorio o permanente^6^. El delirium en Unidad de Cuidado Intensivo Pediátrica (UCIP) se asocia con estancias hospitalarias prolongadas, mayor tiempo de ventilación mecánica, y mayor morbi-mortalidad^6^^,^^7^. Asimismo, los niños que cursan con delirium en UCIP tienen riesgo de presentar a largo plazo discapacidad, deterioro cognitivo, y alteraciones de salud mental como depresión, ansiedad y síndrome post-UCI o síndrome de estrés postraumático post UCI^4^^,^^6^. “Cuanto más larga sea la duración del delirium, más severas serán las consecuencias a nivel cognitivo y de memoria”^6^. Por último, el delirium en UCIP supone un aumento de los costos en salud que puede ser hasta del 85%^4^^,^^5^. Esto hace que prevenir, detectar e intervenir a tiempo el DP sea un punto clave para la recuperación inmediata y un óptimo desarrollo futuro de los niños hospitalizados en UCIP.

El cuidado de enfermería consiste en velar, proteger y promover el bienestar de las personas frente a cierta vulnerabilidad, en este caso el delirium como una alteración del equilibrio neurocerebral^3^, de manera que puedan desarrollar la totalidad de su potencial humano y alcanzar una calidad de vida óptima^8^. En este sentido las intervenciones de enfermería son “cualquier tratamiento basado en el criterio y conocimiento clínico, que realiza un profesional de enfermería para favorecer el resultado esperado del paciente”^9^, es decir, el bienestar de la persona. Estas intervenciones pueden ser: De tratamiento o prevención de la enfermedad; directas o indirectas; e independientes o dependientes^9^.

La mayor parte de la investigación en delirium se ha dado desde la medicina y el cuidado crítico, en contraste, desde la ciencia y disciplina de enfermería la literatura es escasa; por lo tanto, se realiza un scoping review con el objetivo de identificar la literatura disponible respecto al cuidado de enfermería no farmacológico para niños menores de 18 años hospitalizados en UCIP que presentan delirium.

## Materiales y Métodos

De acuerdo con el Instituto Joanna Briggs (JBI) un scoping review consiste en una revisión exploratoria, que permite identificar los tipos de evidencia y de investigación disponible sobre un tema determinado, así como los conceptos clave, límites y vacíos de investigación respecto a ese tema^10^^-^^11^. El JBI propone las siguientes etapas para su desarrollo: Definir el título, establecer el trasfondo, revisar la pregunta y el objetivo de investigación, establecer los criterios de inclusión bajo la estrategia PCC, ejecutar la búsqueda, extraer y clasificar los resultados, realizar la discusión y finalmente establecer las conclusiones^10^^-^^11^.

Este estudio se desarrolló siguiendo las guías propuestas por el JBI^10^^-^^12^ y se llevó un registro detallado de cada paso realizado de la metodología a través de una matriz elaborada en la plataforma de Google Spreadsheets.

La pregunta de investigación fue ¿Cuál es el rol de enfermería frente al delirium pediátrico en niños menores de 18 años hospitalizados en UCIP? Los criterios de inclusión fueron definidos con base en el objetivo del estudio y a partir de la Participantes, Concepto y Contexto (PCC) propuesta por el JBI. Se especifican en la Tabla 1

**Tabla 1:** Criterios de inclusión

Participantes	Niños menores de 18 años hospitalizados en UCIP que presenten delirium.
Concepto	Intervenciones de enfermería frente al delirium pediátrico.
Contexto	Unidad de cuidado intensivo pediátrica.

Se tuvieron en cuenta todo tipo de textos científicos, incluyendo artículos de investigación cuantitativos, cualitativos y mixtos, primarios y secundarios, de todos los niveles de evidencia disponible, así como capítulos de libro y documentos de texto y opinión. Los límites que se definieron para la búsqueda fueron de tiempo, incluyendo solamente publicaciones desde el año 2015 hasta el 2021, y de idioma, que se restringió a inglés, español y portugués.

### Estrategia de búsqueda

Se consultaron 7 bases de datos que fueron WebOfScience, Medline, ScienceDirect, Scielo, Biblioteca Virtual en Salud (BVS), LILACS y OpenGrey. Esta última plataforma fue el recurso utilizado para buscar literatura gris. La ecuación de búsqueda que se utilizó fue (((nursing care) AND (child OR children)) AND (delirium)) AND (Pediatric Intensive Care Unit) con sus variaciones en español y en portugués. La búsqueda se ejecutó entre los meses de febrero y abril del año 2021 y derivó en 153 recursos científicos que fueron filtrados con base en los criterios de inclusión establecidos.

### Selección de evidencia

Después de haber realizado la búsqueda en cada base de datos, los resultados se extrajeron y se cargaron en la plataforma Rayyan, una herramienta web diseñada para gestionar revisiones, de uso libre la cual no requiere licencias^13^. En esta plataforma se identificaron y eliminaron los hallazgos repetidos. Posteriormente se realizó un filtro basado en el título y el abstract de los documentos encontrados para seleccionar aquellos que correspondían a los criterios de inclusión; en caso de que no se pudiera definir claramente se acudió a un tercer revisor y se revisó el texto completo. Esta selección se realizó por dos enfermeras especialistas en cuidado crítico de adulto y pediátrico.

**Figura 1:**
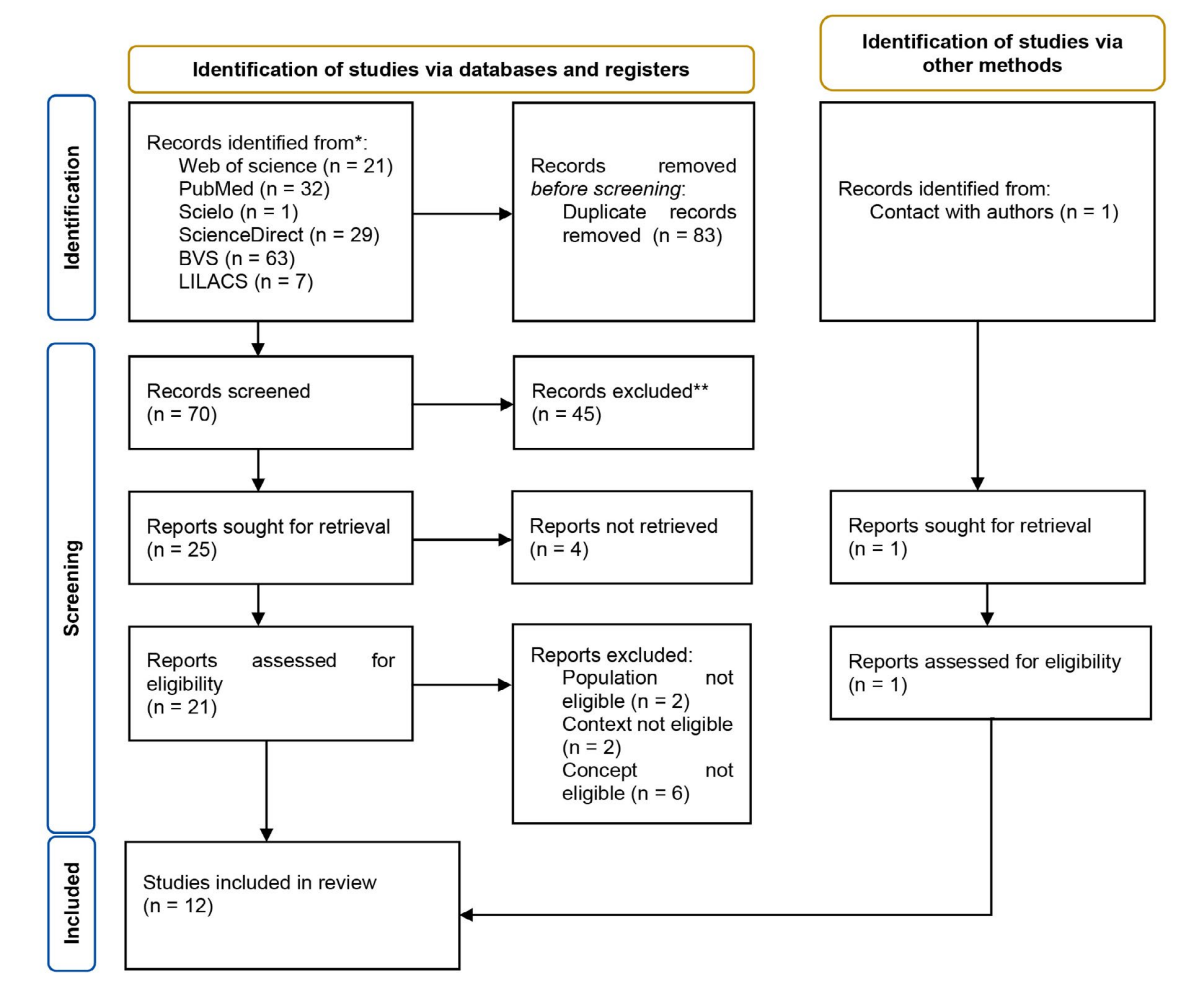
Diagrama de flujo PRISMA.

El diagrama de flujo PRISMA (Figura 1)^14^ sintetiza el proceso que se llevó a cabo para la selección de recursos.

Fuente: Page MJ, McKenzie JE, Bossuyt PM, Boutron I, Hoffmann TC, Mulrow CD, et al. The PRISMA 2020 statement: An updated guideline for reporting systematic reviews. BMJ. 2021;372

### Extracción de datos

Los artículos que quedaron incluidos de forma definitiva fueron revisados por 2 investigadores para la extracción de datos. Se realizó lectura crítica de los artículos utilizando las herramientas para Evaluación Crítica del instituto JBI^15^, disponibles en su página web, para evaluar la calidad del estudio. También se evaluó el nivel de evidencia de la literatura encontrada.

La extracción de datos y el análisis de los recursos finales se hizo a través de una matriz diseñada en Google Spreadsheets en la cual se recopiló la información de identificación del artículo (título, autores, fuente o revista donde fue publicada, país, año, metodología e idioma original), el nivel de evidencia del recurso y la información relevante para este scoping review (población o participantes del estudio, contexto, intervención realizada, resultado, categoría de resultado e información adicional relevante). Después de la lectura, se asignó el estudio a una categoría según la intervención principal realizada por los autores del estudio. La información validada y la base de datos se registró y almacenó en Mendeley Data^16^

## Resultados

Se seleccionaron 12 artículos finales que respondían a la pregunta de investigación y cumplían con los criterios de inclusión, los cuales fueron caracterizados y analizados utilizando la matriz descrita anteriormente.

### Características de los recursos revisados

Los recursos encontrados fueron artículos publicados en revistas de diferentes nacionalidades, entre los cuales se destacaron un estudio transversal de alcance internacional y un estudio realizado a nivel de Europa continental. El idioma original de publicación fue el idioma inglés y el país con más publicaciones fue Estados Unidos de América. La totalidad de las características de los artículos seleccionados se describen en laTabla 2.

**Tabla 2:** Identificación y caracterización de los resultados obtenidos

n	Categoría	Título	Autores	Revista	País	Año	Tipo de estudio	Idioma original	Nivel de evidencia JBI
1	Abordaje investigativo	The Life Course Health Development Model: A Theoretical Research Framework for Pediatric Delirium	Kalvas, LB.^17^	Journal of Clinical Nursing	Reino Unido	2019	Revisión critica	Inglés	Nivel 4.A
2	Intervenciones de educación al personal	Overcoming Barriers to Delirium Screening in the Pediatric Intensive Care Unit	Rohlik, GM, et al.^18^	Critical Care Nurse	Estados Unidos	2018	Observacional analítico	Inglés	Nivel 3.D
3		Delirium Knowledge, Self-Confidence, and Attitude in Pediatric Intensive Care Nurses	Norman, SL, et al.^19^	Journal of Pe diatric Nursing	Estados Unidos	2019	Cuasi experimental	Inglés	Nivel 2.C
4		Assessing Nursing and Pediatric Resident Understanding of Delirium in the Pediatric Intensive Care Unit	McGetrick, ME, et al.^20^	Critical care nursing clinics of North America	Reino Unido	2019	Estudio de casos	Inglés	Nivel 4.C
n	Categoría	Título	Autores	Revista	País	Año	Tipo de estudio	Idioma original	Nivel de evidencia JBI
5	Intervenciones de confort	Pediatric Delirium: Early Identification of Barriers to Optimize Success of Screening and Prevention	Franken, A, et al.^21^	Journal of Pe diatric Health Care	Estados Unidos	2019	Cuasiexperimental	Inglés	Nivel 2.C
6		Management of Pediatric Delirium in Pediatric Cardiac Intensive Care Patients: An International Survey of Current Practices.	Staveski, SL, et al.^22^	Pediatric Critical Care Medicine	Estados Unidos/Internacional	2018	Transversal - Des criptivo	Inglés	Nivel 4.B
7		Quality Improvement Initiative to Reduce Pediatric Intensive Care Unit Noise Pollution With the Use of a Pediatric Delirium Bundle	Kawai, Y, et al.^23^	Journal of Intensive Care Medicine	Estados Unidos	2019	Observacional	Inglés	Nivel 3.C
8	Intervenciones integrales	Delirium in Children: Identification, Prevention, and Management	Bettencourt, A, et al.^24^	Critical Care Nurse	Estados Unidos	2017	Revisión de lite ratura	Inglés	Nivel 5.B
9		Clinical recommendations for pain, sedation, withdrawal and delirium assessment in critically ill infants and children: an ESPNIC position statement for healthcare professionals	Harris, J, et al.^25^	Intensive Care Med	Europa	2016	Guía Basada en la Evidencia	Inglés	Nivel 2.B
10		Pediatric Delirium in the Cardiac Intensive Care Unit: Identification and Intervention.	Bryant, KJ, et al.^26^	Critical Care Nurse	Estados Unidos	2018	Revisión de literatura	Inglés	Nivel 5.B
11		Delirium in paediatrics: early detection, diagnosis and nursing care	Henao- Castaño, A, et al.^27^	Revista Científica de la Sociedad de Enfermería Neurológica	España	2020	Revisión integrativa	Inglés	Nivel 4.A

Con respecto al periodo de tiempo, el estudio más antiguo fue de 2016 y el más reciente de 2021, siendo el 2019 el año con más publicaciones (5 artículos). En cuanto al tipo de estudio se encontraron 7 estudios primarios y 5 estudios secundarios (revisiones de literatura) y el rango de evidencia en el que se encontraron varió desde el nivel 2B hasta el 5B. La Tabla 3 muestra distribución de los artículos encontrados en función de la metodología de investigación y categoría de resultado.

**Tabla 3:** Distribución de los artículos encontrados según la metodología de investigación y categoría de resultado

Tema	Abordaje investigativo	Educación al personal	Intervenciones de confort	Intervenciones integrales	Total
Cuasi experimental		1	1		2
Guía Basada en Evidencia				1	1
Estudio de casos		1			1
Observacional			1	1	2
Observacional analítico		1			1
Revisión crítica	1				1
Revisión de literatura				2	2
Revisión integrativa				1	1
Transversal			1		1
Total	1	3	3	5	12

### Categorías de resultados

Se identificó la intervención principal de cada artículo y posteriormente se agruparon los artículos que tenían intervenciones similares dentro de una misma categoría. Finalmente se establecieron 4 categorías de resultado: Abordaje investigativo, intervenciones de educación al personal, intervenciones de confort e intervenciones integrales. Los estudios se clasificaron de la siguiente manera en esas 4 categorías (Tabla 4).

**Tabla 4:** Distribución de los artículos según categorías de resultado

Cód	Categoría	n artículos
I	Abordaje investigativo	1
II	Intervenciones de educación al personal	3
III	Intervenciones de confort	3
IV	Intervenciones integrales	5
	Total	12

### Abordaje investigativo

Kalvas^17^ realizó un marco teórico para la investigación de DP desde el modelo de desarrollo de la salud a lo largo de la vida (LCHD) el cual puede ser útil para investigar las consecuencias permanentes y las alteraciones a largo plazo que puede causar el delirium sobre la estructura y funcionamiento del cerebro infantil, y el impacto potencial que tiene este síndrome sobre la trayectoria de salud y vida de la población pediátrica que presenta delirium durante la estancia en UCIP. También resalta el liderazgo de enfermería frente a la identificación, prevención y tratamiento del delirium, así como la importancia de las intervenciones de enfermería como los protocolos de sedación, la movilización temprana, la promoción del sueño y la presencia de la familia para disminuir la incidencia y duración del delirium. Asimismo, la autora promueve la implementación y evaluación de estas estrategias.

### Intervenciones de educación al personal

En algunos de los estudios el abordaje al DP inició con intervenciones al personal de la UCI responsable de prevenir, identificar y tratar este síndrome. En este caso se encontró que el tamizaje de delirium mejoró en un 20% después de una intervención educativa para el personal implementada como parte de un programa de mejoramiento de calidad^18^, así como un aumento significativo en el conocimiento, autoconfianza y actitud del personal de enfermería hacia el delirium después de una estrategia educativa^19^.

Adicionalmente es posible que la falta de conocimiento frente al delirium, su prevención, diagnóstico y tratamiento tenga implicaciones negativas sobre el cuidado del paciente, pues conlleva a un déficit en el diagnóstico y el tratamiento^20^.

### Intervenciones de confort

El confort se presenta en las dimensiones física, emocional, social y ambiental, las cuales se interrelacionan entre sí.

Las intervenciones de confort físico incluyen las técnicas de promoción de sueño, técnicas de orientación, vigilancia diaria de catéteres, líneas e inmovilizaciones^21^, masajes, acupuntura, toque sanador^22^, disminución de la sedación, aumento de la movilidad, manejo del dolor, evaluación y retiro de medicación y evitar el uso de fármacos deliriogénicos^23^.

El confort social del paciente se puede mejorar permitiendo la presencia de la familia en la unidad21,22 y educando a la familia y los visitantes sobre el delirium en UCI^23^. Por último, para garantizar el confort ambiental se debe permitir la presencia de objetos familiares en la unidad, alternar ciclos de luz- oscuridad^21^, y disminuir los estímulos visuales y sonoros para mejorar la calidad del sueño^22^^,^^23^. Algunos autores como Franken y Kawai diseñaron paquetes de intervenciones no farmacológicas orientadas al confort del paciente. El paquete utilizado en el estudio de Franken et al.^21^ demostró ser prometedor para la prevención del delirium y disminuyó la incidencia de este síndrome en la UCIP donde se utilizó, sin embargo, los autores no afirman que el uso de estos paquetes disminuya consistentemente la incidencia de delirium. Por otro lado, el paquete Bundle to Eliminate Delirium (BED) diseñado por Kawai et al se centró en la disminución de ruido y mejoramiento de la calidad de sueño en la UCI para disminuir el DP y demostró ser efectivo ya que el ruido disminuyó en 6dB en las unidades donde se implementó^23^.

### Cuidados integrales

El manejo integral de delirium inicia con la identificación y modificación de factores que contribuyen a su desarrollo^24^, es decir que se deben implementar estrategias preventivas antes de que aparezcan los síntomas. En función de esto es importante utilizar instrumentos de valoración de cabecera para evaluar el delirium y realizar valoración y tamizaje para delirium de manera rutinaria al menos una vez por turno, 24-48 horas después del ingreso, o según la condición clínica del niño^25^. Después de que el síndrome se haya instaurado y diagnosticado se debe identificar y resolver la etiología subyacente^24^^-^^26^, controlando el dolor y teniendo en cuenta los cuidados en torno a la farmacoterapia, entre los cuales se encuentran la sedación adecuada, la administración de medicamentos y el manejo de eventos adversos a antipsicóticos^24^^,^^26^^,^^27^. Estas intervenciones deben ir acompañadas de intervenciones de confort^24^^,^^27^.

El cuidado de enfermería varía desde la valoración hasta el apoyo emocional para el paciente y la familia, además requiere conocimiento y entrenamiento en las enfermeras para que sea de calidad^27^. El conocimiento y entrenamiento del personal de enfermería frente al DP es clave, ya que el desconocimiento del personal conlleva a confundir el delirium con otras entidades clínicas y dificulta el diagnóstico y tratamiento^27^.

Algunos autores promueven la implementación del paquete ABCDEF^27^^,^^28^, es un paquete de intervenciones para liberar a pacientes de soporte vital que incluye el manejo de delirium^29^. Esta estrategia ha sido utilizada en adultos, sin embargo, la encuesta de Huang et al. sobre la implementación del paquete ABCDE en UCIP en el sur de China reveló que el uso de este paquete en entornos pediátricos es significativamente menor al que se presenta en unidades de cuidado intensivo de adultos y que además la mitad de los respondientes de este estudio no conocían esta estrategia^28^.

Intervenciones como el paquete ABCDE representan estrategias multicomponente basadas en evidencia para liberar de soporte vital los pacientes hospitalizados en UCI y reducir el delirium y la debilidad; las cuales están interconectadas de modo que unas influyen sobre otras y que una actividad contribuye a diferentes objetivos. Este paquete compendia las siguientes intervenciones^29^^,^^30^:

ABC: Pruebas protocolizadas de respiración espontánea para retirar la sedación y la ventilación mecánica.
C: Coordinación y selección de sedación y analgesia.
D: Monitoreo y manejo de delirium.
E: Ejercicio y movilidad temprana.

Algunos autores también lo han denominado ABCDEF, en donde la F significa involucrar y empoderar a la familia^27^^,^^30^. Esta estrategia fue diseñada inicialmente para retirar la ventilación mecánica (VM) a pacientes críticos hospitalizados en UCI^29^, sin embargo, las intervenciones que reúne aplican para prevenir y manejar el delirium de forma integral, por lo tanto, es fundamental adaptarla al contexto pediátrico.

## Discusión

La literatura disponible respecto a cuidados de enfermería frente al DP en UCIP abarca estudios primarios de tipo observacional, transversal y cuasi experimental, y estudios secundarios como revisiones; así como diferentes niveles de evidencia: Desde el 5B hasta el 2B. Con frecuencia los estudios evalúan intervenciones para manejo de delirium y el conocimiento y las prácticas del personal de UCIP frente a esta entidad clínica.

El cuidado holístico frente al delirium se hace evidente a través de diferentes estrategias directas e indirectas que pueden ser de prevención, diagnóstico o tratamiento. Las intervenciones preventivas son el primer paso para manejar el DP, evitando su aparición. La prevención del DP puede realizarse a partir de la identificación y modificación de factores de riesgo y desencadenantes y del tamizaje periódico de delirium en la UCIP^22^^,^^23^. Asimismo, cuando ya hay delirium, es importante identificar y tratar la causa^24^^-^^26^ y si se llegan a utilizar medicamentos antipsicóticos se deben prevenir, vigilar y manejar los efectos secundarios asociados^24^^,^^26^^,^^27^.

Las intervenciones de confort son un componente importante del cuidado de enfermería frente al DP y deben partir del paciente como centro alcanzando su familia y su entorno. Las medidas se confort físico se enfocan en promover la comodidad del paciente a través de la evaluación de los catéteres y vías, control adecuado del dolor, protocolos de sedación, evaluación y retiro de medicamentos^21^^,^^23^. También se incluyen las técnicas de promoción de sueño y de orientación del paciente. Estas últimas requieren acciones de confort físico en conjunto con medidas de confort ambiental, como permitir que el niño tenga algún objeto conocido o fotos de sus familiares en la unidad, alternar ciclos luz- oscuridad y disminuir la luz y el ruido para mejorar el patrón de sueño^21^^-^^23^.

El confort social y emocional del paciente se fortalecen con la presencia de la familia, por lo tanto, es importante permitir estos encuentros, además de educar a la familia sobre el delirium y darle apoyo emocional^21^^,^^23^^,^^27^.

Otra función de los profesionales de enfermería es la educación al personal frente al DP, pues cuando los profesionales desconocen este síndrome, pueden llegar a confundirlo con otras entidades clínicas lo cual deriva en un diagnóstico o tratamiento ausente o inadecuado y a su vez concluye en resultados desfavorables para la salud del paciente y la calidad del cuidado^20^^,^^24^^,^^27^^,^^28^. Es necesario mejorar la educación sobre el delirium en todos los niveles de formación para mejorar su identificación y tratamiento y al mismo tiempo fortalecer la capacidad y confianza del personal frente al mismo^20^.

La detección y manejo de delirium pediátrico puede verse como una práctica de calidad de la atención en salud^18^^,^^23^ pues es una forma de incidir positivamente sobre los resultados en salud de una población, tanto a corto como a largo plazo y en un enfoque de curso de vida.

Algunas limitaciones de este estudio fueron que no se incluyeron estudios relacionados con validez de escalas de valoración de delirium y que la búsqueda se enfocó mayormente en intervenciones de tratamiento o curativas y en menor medida de prevención y diagnóstico temprano.

## Conclusión

Para abordar el DP de manera efectiva e integral se requiere entender el cuidado con intervenciones de humanización incluidos los aspectos individuales de la persona, su familia, el entorno donde se encuentra hospitalizado y el personal que lo atiende, incidiendo sobre los factores individuales y estructurales que contribuyen a su aparición.

Las intervenciones de confort son las principales intervenciones no farmacológicas, orientadas al confort ambiental, físico, social y emocional del niño. Son fáciles de implementar, constituyen un pilar fundamental para el cuidado de enfermería frente al DP.

La implementación de protocolos o paquetes de intervenciones multicomponente es una estrategia que reúne diferentes actividades de prevención y tratamiento del delirium de manera que facilitan su manejo.

El conocimiento del personal de UCIP sobre el DP es un factor estructural vital que influye sobre la prevención, diagnóstico y tratamiento de este síndrome, permite que el personal tenga liderazgo y confianza en cuanto al manejo de delirium y sustenta las demás intervenciones, por lo tanto, es importante incidir sobre él a través de actividades de educación para el personal.

Existe una necesidad de realizar educación con el personal de UCIP y evaluar su efectividad a largo plazo en términos de reducción de incidencia de delirium, así como incorporar la investigación en la cual el delirium pediátrico sea un indicador de la calidad del cuidado y de la atención en salud.

El cuidado de enfermería oportuno y efectivo frente al DP es esencial para los niños que lo sufren, no sólo porque influye sobre el curso de la enfermedad crítica, sino también porque es una forma de proteger su trayectoria de salud y vida y de promover su desarrollo y calidad de vida óptima.
